# The Mitochondrial Translocator Protein and Arrhythmogenesis in Ischemic Heart Disease

**DOI:** 10.1155/2015/234104

**Published:** 2015-03-30

**Authors:** Lukas J. Motloch, Jun Hu, Fadi G. Akar

**Affiliations:** Cardiovascular Institute, Icahn School of Medicine at Mount Sinai, Hess Center for Science & Medicine, One Gustave L. Levy Place, P.O. Box 1030, New York, NY 1002, USA

## Abstract

Mitochondrial dysfunction is a hallmark of multiple cardiovascular disorders, including ischemic heart disease. Although mitochondria are well recognized for their role in energy production and cell death, mechanisms by which they control excitation-contraction coupling, excitability, and arrhythmias are less clear. The translocator protein (TSPO) is an outer mitochondrial membrane protein that is expressed in multiple organ systems. The abundant expression of TSPO in macrophages has been leveraged to image the immune response of the heart to inflammatory processes. More recently, the recognition of TSPO as a regulator of energy-dissipating mitochondrial pathways has extended its utility from a diagnostic marker of inflammation to a therapeutic target influencing diverse pathophysiological processes. Here, we provide an overview of the emerging role of TSPO in ischemic heart disease. We highlight the importance of TSPO in the regenerative process of reactive oxygen species (ROS) induced ROS release through its effects on the inner membrane anion channel (IMAC) and the permeability transition pore (PTP). We discuss evidence implicating TSPO in arrhythmogenesis in the settings of acute ischemia-reperfusion injury and myocardial infarction.

## 1. Introduction

**Figure 1:**
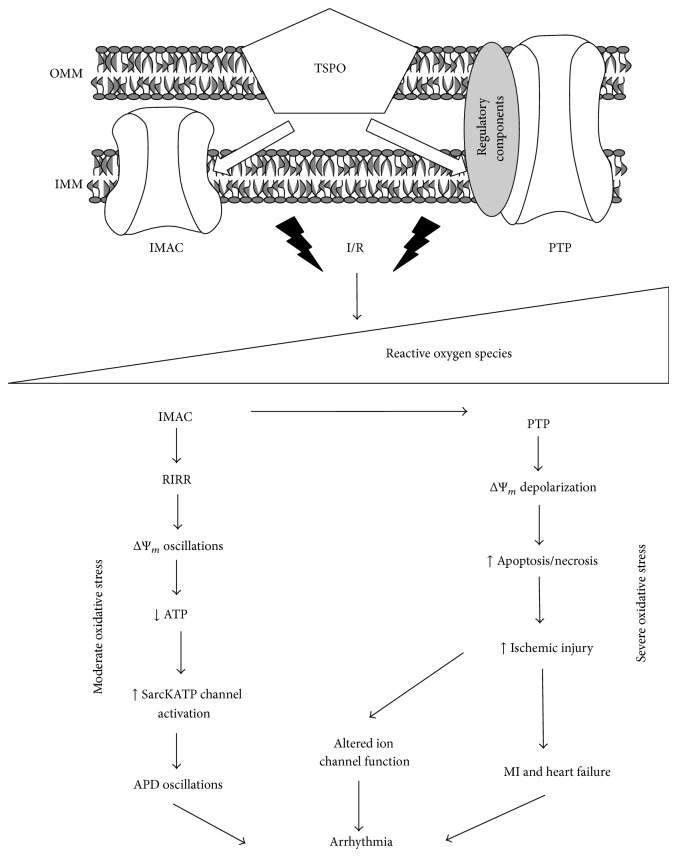
Schematic illustration of the central role of TSPO in the pathogenesis of arrhythmias in response to oxidative stress. APD: action potential duration; IMM: inner mitochondrial membrane; OMM: outer mitochondrial membrane; RIRR: ROS induced ROS release.

The translocator protein (TSPO), formerly known as the peripheral benzodiazepine receptor (PBR), is an 18 kDa mitochondrial protein consisting of 169 amino acids [1]. Arranged in five transmembrane domains, TSPO is a nuclear-encoded protein localized on chromosome 22q13.31 [2–4]. TSPO, which exhibits a highly conserved structure, has been cloned from multiple species including man, dog, cow, pig, rat, and mouse [2, 5]. Enriched in the outer mitochondrial membrane (OMM), TSPO is an integral component of a macromolecular complex of proteins that regulates cell survival and death pathways [1, 2]. Found in most organs within the body, TSPO exhibits robust expression in secretory and glandular tissue, kidney, liver, brain, and heart [1, 2, 6]. The widespread distribution of TSPO is consistent with its diverse physiological functions. These include, but are not limited to, membrane biogenesis, heme biosynthesis, immunomodulation, bioenergetics, redox balance, apoptosis, and cholesterol binding and transport [1, 7–11]. As such, altered TSPO expression and activity in the heart may have important implications for a wide spectrum of cardiovascular disorders. In this review, we highlight the link between TSPO and the pathological process of reactive oxygen species (ROS) induced ROS release (RIRR) which we propose to be a master regulator of electrical dysfunction on one hand and cell death on the other hand. We implicate TSPO in the adverse remodeling associated with ischemia-reperfusion injury and myocardial infarction, both of which are major risk factors for arrhythmias (Figure 1).

## 2. TSPO as a Mediator of ROS Induced ROS Release

Mitochondria synthesize adenine triphosphate (ATP) through oxidative phosphorylation. This highly regulated process is fueled by the mitochondrial membrane potential (ΔΨ_*m*_), which forms the proton-motive force used to shuttle electrons across the electron transport chain [12–14]. In addition to ATP, ROS are also generated when electrons that leak from the ETC combine with oxygen to form superoxide anions (O_2_^−^) [15]. In healthy myocardium, ROS production is countered by efficient antioxidant defense systems which maintain physiological redox balance. In diseased myocardium, overproduction and/or defective scavenging of ROS often leads to oxidative stress (OS). Understanding mechanisms by which myocardial ROS levels are amplified to cause OS is critical for our ability to combat prevalent diseases, in which OS is a hallmark feature.

The concept of RIRR was born from studies by Zorov et al. [16, 17] and Aon et al. [18–20]. These investigators demonstrated that local ROS injury within a discrete region of a cardiomyocyte can rapidly accumulate across a critical mass of the mitochondrial network to cause cellular OS. As such, RIRR describes a fundamental mechanism by which cardiac mitochondria react to elevated ROS levels by stimulating endogenous ROS production. Indeed, this regenerative, autocatalytic process ultimately results in cellular dysfunction and death [17]. Distinct modes of RIRR have been postulated based on their dependence on either the mitochondrial permeability transition pore (PTP) or the inner membrane anion channel (IMAC) [21, 22], both of which are modulated by TSPO. As will be highlighted below, TSPO-mediated regulation of RIRR can influence postischemic arrhythmias directly via IMAC and indirectly via PTP.

## 3. TSPO Regulates RIRR via IMAC

Multiple ROS-sensitive ion channels exist in the mitochondrial membrane. Of key importance is the IMAC which was discovered in liver and heart mitochondria [23]. In a series of seminal studies, Garlid and colleagues demonstrated the anion selectivity of IMAC and its dependence on pH and temperature [23–27]. As with other anion channels, IMAC was readily inhibited by stilbene-22′-disulfonates [28]. IMAC activity was also reduced by Ca^2+^, Mg^2+^ [23, 25–27, 29], and a variety of cationic amphiphilic agents [23, 24]. Studies of drug-channel interactions were consistent with the notion that IMAC mediated the efflux of O_2_^−^ [30, 31]. This, in turn, implicated the channel in the regulation of cellular bioenergetics and redox properties [32]. Although the molecular structure of IMAC remained elusive, its tight regulation by TSPO-acting ligands (4′ClDzpm, PK11195, and IX protoporphyrin) suggested a close interaction between its pore-forming subunit in the inner mitochondrial membrane (IMM) and the regulatory protein TSPO in the OMM [12, 24, 33]. Despite these studies, the nonspecific effects of many TSPO ligands on calcium handling and other cellular processes such as contractility and excitability have confounded our ability to pinpoint the direct role of TSPO per se in cardiac pathophysiology [34–38]. Nonetheless, studies by O'Rourke and colleagues have elegantly documented the role of IMAC in RIRR-mediated metabolic and electrophysiological instabilities. Indeed, these investigators demonstrated that photo-induced oxidation of a discrete region within the cardiac myocyte can unleash a regenerative process of ETC-derived ROS that was dependent on IMAC. Once ROS levels across a critical portion (60–70%) of the mitochondrial network exceeded a given threshold, sustained ΔΨ_*m*_ oscillations were initiated. This highly nonlinear property was coined as “mitochondrial criticality” [20, 39]. Using computational modelling, Cortassa and colleagues demonstrated that the frequency of the synchronized cell-wide mitochondrial oscillations was strongly modulated by ROS scavengers and the rate of oxidative phosphorylation [40]. Importantly, RIRR-evoked mitochondrial oscillations gave rise to cellular electrophysiological oscillations that were dependent on the cyclical activation of sarcolemmal K_ATP_ channels. Activation of these normally dormant channels is thought to be protective as they act to preserve energy at a time of increased metabolic demand. However, increasing K efflux through these channels can induce rapid and heterogeneous action potential duration (APD) shortening and suppress myocyte excitability in a manner that predisposes to reentrant arrhythmias [12, 13, 39, 41]. Importantly, both metabolic and electrophysiological oscillations could be readily abolished by TSPO ligands, which functionally reduce cardiomyocyte ROS levels [18–20, 39, 42].

More recently, we extended the concept of RIRR from a subcellular phenomenon to one occurring at the organ level. Using optical mapping approaches, we demonstrated the functional significance of RIRR in terms of arrhythmia propensity [42–45]. In a model of moderate OS produced by relatively brief challenge with H_2_O_2_ perfusion, TSPO inhibition abolished the large amplitude secondary O_2_^−^ peak which arose following, not during, the exogenous oxidative challenge [43]. Prevention of this secondary O_2_^−^ peak abrogated ventricular fibrillation and suppressed the frequency of arrhythmogenic triggers [43]. Indeed, these findings highlighted the importance of IMAC-mediated O_2_^−^ release as a driving force for RIRR associated arrhythmias in intact myocardium.

## 4. TSPO May Regulate RIRR via PTP

In addition to IMAC, the PTP is also activated in response to rising ROS levels [21, 46]. However, a hierarchal pattern seems to govern the activation sequence of the two channels [18]: IMAC is activated firstly in response to moderate levels of OS followed by the activation of the nonspecific, high-conductance PTP. Despite considerable debate over its molecular composition, the PTP is strongly regulated by the voltage-dependent anion channel (VDAC) in the OMM, the adenine nucleotide translocase (ANT) in the IMM, and Cyclophilin D in the mitochondrial matrix [46]. Importantly, the Bernardi group has recently demonstrated the role of dimers of the ATP synthase in the formation of the PTP [47]. A causative relationship between OS-induced mitochondrial permeability transition and ΔΨ_*m*_ depolarization has also been demonstrated in numerous studies. Zorov et al. [16, 17] found a direct correlation between PTP activation and myocyte death. These findings, combined with evidence that ΔΨ_*m*_ depolarization was abolished by the Cyclophilin D inhibitor Cyclosporin A (CsA), suggested an important role for PTP-mediated ΔΨ_*m*_ depolarization in RIRR-mediated apoptosis [16].

We recently investigated the efficacy of the PTP desensitizer CsA in protecting against OS-induced mitochondrial and electrical dysfunction at the intact heart level [48]. Unlike our previous study [43], we chose a severe model of continuous H_2_O_2_ challenge which reliably and predictably caused irreversible ΔΨ_*m*_ depolarization within a 30 min time-frame, likely through PTP activation [48]. Our experiments uncovered a dual role for CsA in either protecting or impairing cardiac function depending on the cellular milieu [48]. Specifically, we found that CsA-mediated cardioprotection in this severe model of oxidative challenge required mitochondrial K_ATP_ (mK_ATP_) channel activation through a protein kinase C dependent pathway [48]. Increasing mK_ATP_ activity during CsA administration was required for limiting OS-induced electromechanical dysfunction. On the other hand, CsA administration during conditions that prevented mK_ATP_ channel activation exerted unintended proarrhythmic consequences possibly through accelerated APD shortening [48]. Our findings addressed existing controversy in the basic and clinical literature surrounding the utility of CsA as a cardioprotective agent [48–53].

In addition to Cyclophilin D, there is some evidence that the PTP may also be regulated by TSPO through interaction with the VDAC and ANT [54–56]. This area, however, requires further clarification considering the elegant findings of Šileikyte et al. who recently discounted a major role of TSPO in the regulation of the PTP by the outer membrane [57]. As such, more studies aimed at defining the macromolecular complex of proteins that forms the PTP are critical to our ability to identify new therapeutic targets. This is especially important considering the apparent shortcomings of CsA, which fails to abrogate laser flash-induced ΔΨ_*m*_ oscillations [19] and promotes rather than prevents arrhythmias during conditions that prohibit mK_ATP_ channel activation [48]. Indeed, novel approaches aimed at limiting ROS-mediated PTP opening are likely to have major clinical implications. As such, targeting regulatory PTP components via TSPO may have a dual beneficial impact by improving electrical function while simultaneously promoting cell survival in response to OS [54, 55]. Using the TSPO ligands SSR180575 and 4′ClDzp, Leducq et al. successfully inhibited ΔΨ_*m*_ depolarization in isolated cardiac mitochondria after excessive ROS exposure. In addition, both agents reduced apoptosis linked events, including cytochrome c release, caspase-3 activation, and DNA fragmentation [58].

## 5. Role of TSPO in Postischemic Arrhythmias

Ischemic heart disease is a major public health epidemic and a leading cause of morbidity and mortality worldwide [59, 60]. Ischemic injury predisposes to myocardial infarction, heart failure, arrhythmias, and sudden cardiac death. Prompt restoration of oxygenated blood flow to the ischemic myocardium is required for limiting the extent of irreversible cell damage and death [61]. Unfortunately, restoration of blood flow, in itself, results in additional cardiac damage known as reperfusion injury [62]. Such ROS-mediated damage is more severe when reperfusion therapy is delayed. Reperfusion-mediated redox imbalance and cytosolic calcium overload promote mechanoelectrical dysfunction and the genesis of lethal arrhythmias shortly upon reperfusion.

Although earlier studies showed that TSPO-acting ligands which reduced ROS levels were effective in abolishing metabolic and electrophysiological oscillations, their impact on arrhythmias in clinically relevant scenarios was not established until more recently [42]. We investigated whether protection against mitochondrial depolarization could translate into an antiarrhythmic benefit in response to ischemia-reperfusion injury. IMAC blockade blunted ischemia-induced APD shortening and the onset of inexcitability in a dose-dependent manner [42]. In contrast, IMAC activation using FGIN-1-27 accelerated APD shortening and resulted in an early form of conduction failure during ischemia [42]. Specifically, hearts that underwent IMAC activation prior to ischemia exhibited heightened sensitivity to ischemia. Using high-resolution optical mapping, we identified discrete areas of conduction block in these hearts during early ischemia that persisted upon reperfusion and likely promoted the formation of reentrant activity underlying postischemic ventricular fibrillation [42]. Remarkably, IMAC blockade, which stabilizes ΔΨ_*m*_ *in vitro,* suppressed these arrhythmias and promoted the rapid recovery of the action potential upon reperfusion [42]. The protective effect of IMAC blockade on postischemic electrical function was also evident in a rabbit model of ischemia-reperfusion injury [35]. Of note, the antiarrhythmic effect afforded by TSPO ligands was not evident in hearts treated with the PTP desensitizer, CsA. This reinforces IMAC as a primary mitochondrial mediator of acute postischemic arrhythmias.

## 6. Role of TSPO in Myocardial Infarction

Myocardial infarction (MI) is a global health epidemic that predisposes to heart failure and arrhythmias [60]. Indeed, the majority of cardiac-related deaths occur in patients who develop MI as a consequence of coronary artery disease [63]. In those patients the risk of cardiac arrest is approximately 4–6 times that of the general population [64]. PTP opening in response to severe ischemia-reperfusion injury results in MI as a consequence of myocyte loss to necrosis/apoptosis [46]. Since MI is a major risk factor for heart failure and arrhythmias, PTP inhibition may exert an indirect antiarrhythmic effect by reducing infarct size and improving overall cardiac function [65]. In a small placebo-controlled trial involving 58 STEMI (ST segment elevation myocardial infarction) patients, CsA administration was associated with smaller infarcts at the time of reperfusion [66]. Despite these encouraging clinical findings, the efficacy of CsA in preventing arrhythmias remained unclear [49, 51, 53]. In fact, several experimental, preclinical [52], and clinical findings [50] have cast serious doubts regarding the overall utility and safety profile of CsA. Hence, new approaches for combatting MI are urgently needed.

TSPO ligands inhibit PTP function through a distinct mechanism of action that is not dependent on Cyclophilin D [54, 56, 67]. As such, these agents may be an exciting alternative to CsA. Indeed, SSR180575 improved left ventricular function and reduced infarct size in rat and rabbit models of ischemia-reperfusion injury [58]. Moreover, TSPO inhibition with 4′ClDzp improved the rate of oxidative phosphorylation, inhibited PTP opening, and limited the rate of apoptosis in isolated mitochondria [67]. Interestingly, the cardioprotective efficacies of 4′ClDzp and PK11195 were similar to those elicited by ischemic preconditioning or diazoxide treatment [67]. Similar results were obtained by Xiao et al. [68] who observed increased ETC activity and reduced ROS levels. These favorable bioenergetic properties were associated with improved recovery of left ventricular mechanical function and a diminished rate of arrhythmias upon reperfusion [68]. Initial results in large animals were also encouraging. Specifically, treatment of pigs with 4′ClDzp at the time of reperfusion following 60 minutes of left anterior descending coronary artery occlusion was associated with rapid ST segment resolution and a trend towards reduced infarct size in the absence of major hemodynamic complications [69].

Recently, the effects of a new TSPO ligand TRO40303 which binds at the cholesterol site of the mitochondrial protein were examined. As with 4′ClDZP, TRO40303 inhibited PTP opening and apoptosis in response to simulated ischemia-reperfusion or hydrogen peroxide challenge in adult or neonatal cardiomyocytes, respectively [70]. These cellular effects correlated with reduced ROS levels and infarct size in anesthetized rats [70]. The efficacy of TRO40303 even when delivered as a single bolus injection before reperfusion provided the impetus needed for testing in humans. In a Phase I double-blind placebo-controlled clinical trial involving 72 volunteers [71], TRO40303 met the safety criteria. This naturally paved the way for a Phase II trial [71, 72]. MITOCARE is an ongoing multicenter, randomized, double-blind, placebo-controlled survey evaluating the safety and efficacy of TRO40303 for the reduction of reperfusion injury in patients undergoing percutaneous coronary intervention for acute MI [72].

Although TSPO-mediated PTP inhibition may be of major therapeutic value in the future, this strategy is complicated by several issues. Of note, the use of higher concentrations of TSPO ligands (i.e., >100 uM 4′ClDZP and >40 uM PK11195) can elicit cardiotoxic effects such as mitochondrial swelling [67, 73, 74]. This highly undesirable property, which is consistent with PTP activation rather than inhibition, raises important questions regarding the nature of the regulation of the PTP by TSPO. On one hand, TSPO-mediated IMAC activation induces O_2_^−^ outflow from the mitochondrial matrix. If this outflow is large enough, it stimulates the regenerative process of RIRR that culminates in mitochondrial depolarization and PTP opening. On the other hand, low levels of IMAC activity may mediate a protective mechanism that limits the build-up of mitochondrial ROS levels required for PTP opening. As such, complete IMAC inhibition using high doses of TSPO ligands may paradoxically accelerate rather than prevent PTP formation. Determining whether the cytotoxic effects observed using high concentrations of these ligands are a consequence of natural cross talk between the PTP and IMAC or due to nonspecific effects of these agents will be of major importance going forward. Finally, TSPO can regulate PTP opening independently of RIRR. By modulating mitochondrial cholesterol uptake and the subsequent generation of oxyterols [75], TSPO ligands can exert additional cardioprotective effects. Taken together, these studies implicate PTP inhibition in the beneficial (namely, infarct sparing) effects of TSPO-acting ligands that reduce ROS levels. This important property is expected to significantly reduce the propensity for arrhythmias by reducing the size of the infarct and slowing the progression towards heart failure.

## 7. Summary and Future Directions

Mounting evidence implicates TSPO in OS-related dysfunction, including arrhythmogenesis. Of key importance is the putative ability of TSPO to modulate two prominent energy dissipating channels, namely, IMAC and the PTP. In doing so, TSPO ligands have shown considerable promise in combatting postischemic ventricular fibrillation (via IMAC inhibition) and MI (via PTP inhibition).

Despite the apparent success of TSPO antagonists in multiple experimental settings, major concerns exist. Of note, high concentrations of TSPO ligands can paradoxically activate the PTP in isolated mitochondria [67, 73, 74], either by disrupting the cross talk between the PTP and IMAC or by eliciting nonspecific effects. Indeed, there is substantial evidence that these agents affect sarcolemmal ion channels, such as the L-type calcium current, even at baseline [35, 38, 76–78]. This, in turn, alters excitation-contraction coupling and contractility. Moreover, findings that 4′ClDzp results in decreased APD in rabbit [35], unchanged APD in guinea pig [42], and prolonged APD in rat (our own unpublished observations) are consistent with an effect on the transient outward K current. Indeed, species-dependent changes in cardiac inotropy and chronotropy by available TSPO ligands further confound the widespread translation of these agents to the clinic [34–38]. Finally, the wide distribution of TSPO receptors in the body presents an added challenge for pharmacological approaches that would likely exert numerous extracardiac effects.

Considering the limitations of currently available TSPO ligands, new biological approaches for modulating TSPO expression and activity in the heart are needed. One such approach is the use of cardiac-specific adeno-associated viral vector serotypes carrying silencing RNAs against TSPO. Indeed, the future design and implantation of such gene therapy approaches may revolutionize the treatment of a host of common cardiovascular disorders in which mitochondrial dysfunction is a hallmark mechanism. In the meantime, a more systematic understanding of the role of TSPO in cardiovascular health and disease is needed.

## Abbreviations

APD: Action potential duration
ANT: Adenine nucleotide translocase
ATP: Adenine triphosphate
CsA: Cyclosporin A
DIDS: Stilbene-22′-disulfonates
ETC: Electron transport chain
IMAC: Inner membrane anion channel
IMM: Inner mitochondrial membrane
OMM: Outer mitochondrial membrane
OS: Oxidative stress
PBR: Peripheral benzodiazepine receptor
PTP: Permeability transition pore
RIRR: ROS induced ROS release
TSPO: Translocator protein
VDAC: Voltage-dependent anion channel.
